# Supplementary material for: Effectiveness of Semi-Supervised Active Learning in Automated Wound Image Segmentation
Source: Int J Mol Sci. 2022 Dec 31;24(1):706. doi: 10.3390/ijms24010706 (PMC9821322; doi:10.3390/ijms24010706)
Supplement: Supplementary file 1 [file ijms-24-00706-s001.zip › ijms-2083031-supplementary.pdf]

Supplementary Materials

A second validation was performed on the Medetec Wound Dataset [31] (MWD): the same dataset was used in the work of Wang et al. and it constitutes a second benchmark for our model. We used the version of MWD provided by Wang et al., which includes 38 images augmented with all possible flips, for a total of 152 samples. Also in this case, the resolution of the photos is more than 2x lower. The comparison of the results obtained by our U-Net model (trained on the *Deepskin* dataset) and the MobileNetV2 model of Wang et al. (trained on the FUSC dataset) is shown in Table S1.

	**Mobile NetV2**	**Our U-Net**
DSC	0.96	0.75
Precision	0.97	0.97
Recall	0.94	0.60
	MWD	

Table S1. Comparison of the results obtained by the MobileNetV2 model (Wang et al.) and the U-Net model proposed in this work, on the MWD dataset. We re-trained the MobileNetV2 on the FUSC dataset for the reproducibility of Wang et al. results. The U-Net model was trained only on the Deepskin dataset as described in the Methods section. For both the models the MWD dataset can be considered as validation set.
